# VO_2_FITTING Software: New Insights and Practical Applications (VO_2_FITTING Software Update)

**DOI:** 10.5114/jhk/194124

**Published:** 2024-12-19

**Authors:** Ana Sofia Monteiro, Rui M. S. Azevedo, Rodrigo Zacca, Anna Ogonowska-Slodownik, Cosme F. Buzzachera, João Paulo Vilas-Boas, Ricardo J. Fernandes

**Affiliations:** 1 Centre of Research, Education, Innovation and Intervention in Sport and Porto Biomechanics Laboratory, Faculty of Sport, University of Porto, Porto, Portugal.; 2 Associate Laboratory i4HB—Institute for Health and Bioeconomy, University Institute of Health Sciences—CESPU, Gandra, Portugal.; 3 UCIBIO—Applied Molecular Biosciences Unit, Forensics and Biomedical Sciences Research Laboratory, University Institute of Health Sciences (1H-TOXRUN, IUCS-CESPU), Gandra, Portugal.; 4 Research Centre in Physical Activity, Health and Leisure, Faculty of Sport, University of Porto, Porto, Portugal.; 5 Laboratory for Integrative and Translational Research in Population Health, Porto, Portugal.; 6 Faculty of Rehabilitation, Jozef Pilsudski University of Physical Education in Warsaw, Warsaw, Poland.; 7 Department of Public Health, Experimental and Forensic Medicine, University of Pavia, Pavia, Italy.

**Keywords:** software, oxygen uptake, off-transient kinetics modelling, symmetry, swimming

## Abstract

This study aimed to present an updated version of VO_2_FITTING software, where it is possible to dynamically edit, process, filter and model $\dot{V}$O_2_ post-exercise data and to characterize the $\dot{V}$O_2_ on/off symmetry along different exercise intensity domains. Validation datasets were developed and applied to four widely used models for describing low, moderate, heavy and severe intensity transitions. Perfect fits were observed and parameter estimates perfectly matched the known inputted values for all available models (standard error = 0; p < 0.001). In addition, an experiment with 10 trained swimmers performing a 5 x 200 m front crawl protocol (with 0.05 m•s^−1^ velocity step increments and 3-min passive rest intervals) was conducted. The on- and off-transient phases were symmetrical in their shape since they were both adequately fitted by a mono-exponential regression model and no slow component was observed independently of the intensity domain. Furthermore, the mono-exponential model without time delay best fitted the $\dot{V}$O_2_ off-transient data. The mean time constant of the on-transient period value was lower than the respective off-transient for all the intensities (15.8 ± 11.4 vs. 30.8 ± 10.4, 11.3 ± 2.3 vs. 29.7 ± 8.4, 13.9 ± 7.0 vs. 28.7 ± 10.8 and 10.3 ± 4.6 vs. 37.0 ± 9.2 s; p < 0.05). VO_2_FITTING is valid, free and open-source software for characterizing $\dot{V}$O_2_ kinetics during both the exercise and recovery periods, helping researchers to give rapid feedback also about the off-transient kinetic parameters.

## Introduction

The assessment of oxygen uptake ($\dot{V}$O_2_) kinetics and the interpretation of its parameters allow the quantification of the physiological mechanisms responsible for the dynamic $\dot{V}$O_2_ response to exercise (on-transient kinetics) and its subsequent recovery (off-transient kinetics) (Jones and Poole, 2013; Özyener et al., 2001). It enables a non-invasive assessment of the control mechanisms of muscle energetics and oxidative metabolism (Jones and Poole, 2013; Rossiter et al., 2005) and, consequently, the effectiveness of a training program, providing relevant information about the exercise tolerance determinants (Poole and Jones, 2012; Zacca et al., 2019). Despite the importance of maximal $\dot{V}$O_2_ ($\dot{V}$O_2max_) for training control and prescription (Duquette and Adam, 2024), the interpretation of $\dot{V}$O_2_ kinetic parameters help researchers and coaches to better understand the responsiveness to training stimuli, particularly regarding the physiological significance of the fast and slow components of the dynamic $\dot{V}$O_2_ response (Burnley and Jones, 2007; Fernandes et al., 2024).

Following the onset of a specific exercise intensity, $\dot{V}$O_2_ on- and off-transient kinetics may have different profiles. Below and at the anaerobic threshold (AnT), i.e., at low and moderate intensities, the $\dot{V}$O_2_ profile, after an initial rise that usually lasts ~15–20 s (phase I or cardiodynamic phase), is described by a mono-exponential function, where an exponential increase is visible (phase II or fast component), followed by a steady-state (phase III) (Monteiro et al., 2020; Özyener et al., 2001; Poole and Jones, 2012). At the recovery period after these exercise intensity domains, $\dot{V}$O_2_ presents a similar behaviour, i.e., a rapid decrease until reaching the baseline values. Above the AnT, at the heavy intensity domain, $\dot{V}$O_2_ response profile starts to differ from less stressful intensities, being usually described by a bi-exponential function (Özyener et al., 2001; Reis et al., 2012). Here, a second $\dot{V}$O_2_ elevation is observed after phase II (after ~90–120 s), known as a $\dot{V}$O_2_ slow component (Billat, 2000; Burnley and Jones, 2007), until a delayed steady-state or exhaustion are attained. Different behaviours of the off-transient kinetics have been described, since it is identified by both mono- and bi-exponential functions (Monteiro et al., 2020; Özyener et al., 2001; Pelarigo et al., 2017). At the severe intensity domain, a bi-exponential function is typically observed both during on- and off-transient kinetics, with a $\dot{V}$O_2_ slow component with significant amplitude (Jones and Poole, 2013; Sousa et al., 2015; Zacca et al., 2019).

Usually, $\dot{V}$O_2_ kinetics has been analysed through mathematical modelling (both for on- and off-transient kinetics) using complex programs, that requires some mastery beyond the knowledge of respiratory physiology (de Jesus et al., 2015; Özyener et al., 2001; Sousa et al., 2011). Considering the importance of giving rapid feedback from experimental testing, it became relevant to create a tool for effective and straightforward analysis of the $\dot{V}$O_2_ response during exercise. Therefore, VO_2_FITTING, a validated, free and open-source software, was developed to characterize $\dot{V}$O_2_ kinetics during continuous exercise (e.g., running, cycling or swimming), allowing to dynamically edit, process, filter and model the typical $\dot{V}$O_2_ responses (Zacca et al., 2019). However, it did not include the possibility of analysing the exercise $\dot{V}$O_2_ off-transient kinetics that can provide additional information on gas exchange dynamics, aiding to better interpret the physiological events supporting the $\dot{V}$O_2_ on-transient response (Jones and Poole, 2013).

The $\dot{V}$O_2_ off-transient kinetics also becomes very useful when the backward extrapolation method is applied, e.g., allowing swimmers to perform without a breathing mask and perform flip turns, achieving competitive velocities, since the testing environment is more ecological (Ribeiro et al., 2016). The aim of the current study was to present an updated version of VO_2_FITTING software that allows to dynamically process $\dot{V}$O_2_ post-exercise data of a large spectrum of exercise intensities, demonstrating the possibility to equally analyse and model $\dot{V}$O_2_ recovery data. In addition, we aimed to contribute to the existent knowledge about the $\dot{V}$O_2_ on/off symmetry by directly comparing the exercise and its subsequent response at low, moderate, heavy and severe intensity domains. We hypothesized that an on/off symmetry would be observed at low and moderate intensities, while above the AnT, i.e., at heavy and severe domains, different exponential models for the exercise and recovery phases would be evidenced.

## Methods

### Development and Validation of VO_2_FITTING Software for Post-Exercise Data Analysis

The already available VO_2_FITTING software (Zacca et al., 2019) was extended for $\dot{V}$O_2_ off-transient kinetics analysis and a swimming experiment was conducted to illustrate how it can be used to edit, process, filter and model the $\dot{V}$O_2_ post-exercise data. The respective installation instructions and other documentation are available at https://shiny.cespu.pt/vo2_news/, and the corresponding author can be reached for further follow-up. Validation $\dot{V}$O_2_ datasets were developed for post-exercise data involving two mono- and two bi-exponential widely used mathematical models for describing different intensity transitions (Özyener et al., 2001; Pelarigo et al., 2017; Sousa et al., 2015). $\dot{V}$O_2_ was used in a raw form as input, without any filtering or processing, and the models were applied without any parameter constraints.

### Participants

Ten trained swimmers (five male) voluntarily participated in the current study, all being engaged in ≥ five swimming training sessions per week. Their main physical characteristics were 16.1 ± 1.7 vs. 15.3 ± 1.2 years of age, 64.0 ± 6.6 vs. 56.5 ± 6.8 kg of body mass and 174.8 ± 4.6 vs. 163.5 ± 6.0 cm of body height for male and female swimmers, respectively, and 495 ± 80 World Aquatics swimming points of best actual competitive performance at the 400 m freestyle event. Swimmers were informed about the purpose of the evaluations and individual written informed consent was provided before data collection, which was approved by the Institutional Ethics Committee of the Faculty of Sport of the University of Porto (protocol code: CEFADE 25 2020; approval date: 11 November 2020) and performed in accordance with the Declaration of Helsinki.

### Design and Procedures

In a 25-m indoor swimming pool (with 27ºC water temperature), and after a 600-m low-to-moderate intensity warm-up, swimmers performed a 5 x 200 m front crawl incremental protocol with 0.05 m•s^−1^ velocity increments and 3-min passive rest intervals between steps (Carvalho et al., 2020; Fernandes et al., 2011; Monteiro et al., 2020). The velocity of the last step was calculated according to each swimmer’s 400 m front crawl time, with a 0.83-s per turn adjustment (due to the use of the respiratory snorkel) (Ribeiro et al., 2016). Subsequently, four successive velocity increments were subtracted to define the subsequent step paces. Velocity was controlled by a visual pacer with flashing lights in the bottom of the pool (Pacer2Swim, KulzerTEC, Aveiro, Portugal) and measured with a manual stopwatch (Seiko, Tokyo, Japan). In-water starts and open turns without underwater gliding were used as previously described (Monteiro et al., 2020).

Pulmonary gas exchange and ventilation were continuously measured breath-by-breath using a portable gas analyser (K4b^2^, Cosmed, Rome, Italy) suspended on a steel cable above the water surface and connected to the swimmer by a low hydrodynamic resistance respiratory snorkel and valve system (Aquatrainer®, Cosmed, Rome, Italy). The respiratory variables were continuously monitored for 3 min during the recovery period (Ribeiro et al., 2016). The gas analysis system and the turbine volume transducer were calibrated before the experiments (following the manufacturer instructions) with gases of known concentrations (16% O_2_ and 5% CO_2_) and a 3-L syringe (respectively). Lactate concentration [La^−^] values were obtained using capillary blood samples from the swimmers’ fingertip at rest, immediately after each step and at the 1^st^, 3^rd^, 5^th^ and/or 7^th^ min post-protocol until reaching the maximal individual value (Lactate Pro2; Arkay Inc., Kyoto, Japan) (Carvalho et al., 2020; Monteiro et al., 2023).

$\dot{V}$O_2_ data were analysed for each incremental protocol step and categorized as low, moderate, heavy and severe intensity domains according to the intensities corresponding to the AnT and $\dot{V}$O_2max_ (Fernandes et al., 2024). To this end, the lactate-velocity curve modelling method was used to determine the interception point of a combined linear and exponential pair of regressions (Carvalho et al., 2020), while conventional physiological criteria were applied to establish $\dot{V}$O_2max_ (de Jesus et al., 2015; Howley et al., 1995). Therefore, the low and moderate exercise domains were identified as corresponding to the step below and the step at the AnT (respectively), and the heavy and severe domains as corresponding to the step below and the step where $\dot{V}$O_2max_ was elicited (respectively) (de Jesus et al., 2015; Pelarigo et al., 2017).

$\dot{V}$O_2_ off-transient kinetic parameters were estimated by bootstrapping and the goodness of fit of each model was analysed with raw data by only excluding errant breaths (Lamarra et al., 1987; Sousa et al., 2015). The off-transient for each intensity domain was estimated with two mono- and two bi-exponential models (Equations 1–4, respectively), and the one that best fitted the data by presenting lower standard error of regression was selected:

1 $${\dot{\text{V}}\text{O}}_{2}\left(\text{t}\right)={\text{EE}\dot{\text{V}}\text{O}}_{2}-{\text{A}}_{\text{p}}\left(1-{\text{e}}^{-\left(t-{\text{TD}}_{\text{P}}/\tau_{\text{P}}\right)}\right),{\text{TD}}_{\text{P}}=0$$

2 $${\dot{\text{V}}\text{O}}_{\text{2}}{\text{(t) = EE}\dot{\text{V}}\text{O}}_{\text{2 }}{\text{– H(t – TD}}_{\text{p}}{\text{)A}}_{\text{p}}{\text{(1 – e}}^{-\text{t – TDp) / τp}}\text{)}$$

3 $${\dot{\text{V}}\text{O}}_{\text{2}}{\text{(t) = EE}\dot{\text{V}}\text{O}}_{\text{2}}\;-\text{ H(t }-{\text{ TD}}_{\text{p}}{\text{)A}}_{\text{p}}\text{(1}-{\text{e}}^{{\text{-t-TD}}_{\text{P}})/\tau_{\text{p}}}\text{) }-\text{H}\left(\text{t}-{\text{TD}}_{\text{SC}}\right){\text{A}}_{\text{SC}}\left(1-e^{-\left(\text{t}-{\text{TD}}_{\text{SC}}\right)/\tau_{\text{SC}}}\right)$$

4 $${\dot{\text{V}}\text{O}}_{\text{2}}{\text{(t) = EE}\dot{\text{V}}\text{O}}_{\text{2}}\;-\text{ H(t }-{\text{ TD)A}}_{\text{p}}\text{(1}-{\text{e}}^{\text{(-t-TD})/\tau_{\text{p}}}\text{) }-\text{H}\left(\text{t}-{\text{TD}}_{\text{SC}}\right){\text{A}}_{\text{SC}}\left(1-e^{-\left(\text{t}-{\text{TD}}_{\text{SC}}\right)/\tau_{\text{SC}}}\right)$$

where $\dot{V}$O_2_(t) (mL•kg^−1^•min^−1^) is $\dot{V}$O_2_ normalized to the body mass at the time t, EE$\dot{V}$O_2_ is the end-exercise $\dot{V}$O_2_ value, A_p_ and A_sc_ (mL•kg^−1^•min^−1^), TD_p_ and TD_sc_ (s), and τ_p_ and τ_sc_ (s) are the amplitudes, time delays and time constants of the fast and slow $\dot{V}$O_2_ components, respectively (Özyener et al., 2001; Sousa et al., 2015), and H represented the Heaviside step function (Ma et al., 2010). To characterize the on/off symmetry response at the different swimming intensity domains, $\dot{V}$O_2_ on-transient kinetic parameters were also estimated using VO_2_FITTING by choosing the model that best fitted to the different intensity swimming efforts from those described in the literature (de Jesus et al., 2015; Özyener et al., 2001; Zacca et al., 2019).

### Statistical Analysis

Noisy (gaussian) and non-noisy validation datasets were developed for the four above-referred models to describe the different intensity transitions. Subsequently, $\dot{V}$O_2_ data as a function of time were uploaded into the software, verifying whether the fitted parameters perfectly matched the known input values. For the experimental study, a post-hoc power calculation indicated that a sample size of 10 subjects with a large effect size would result in a statistical power of 75% (α = 0.05; G*Power 3.1.9.7; Heinrich Heine Universität Düsseldorf, Düsseldorf, Germany). Bootstrapping with 1000 samples was employed to estimate the parameters of mono- and bi-exponential fitting models (a feature available in VO_2_FITTING). When multiple models were applied to $\dot{V}$O_2_ data, an ANOVA F-test was conducted to verify the goodness-of-fit. The mean, standard deviation and the coefficient of variation were calculated for each parameter estimated. A Student’s paired *t*-test was conducted to test for differences between the on- and off-transient kinetic parameters within each intensity domain and between consecutive intensities, using Cohen’s *d* standardized effect sizes (ES) and 95% confidence intervals (CI). For the three comparisons between consecutive intensities, a Hochberg (step-up) correction was used to reduce the type I errors (Menyhart et al., 2021). Furthermore, linear regression and Pearson’s correlation (r) and determination (r^2^) coefficients were also used to assess the relationships between the considered variables. These statistical analyses were conducted using SPSS (version 29.0.0.0; IBM Corporation, Armonk, NY, USA) with a significance level of 5%.

## Results

**Figure 1:**
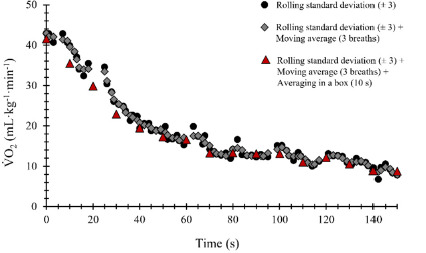
Example of oxygen uptake ($\dot{V}$O_2_) recovery curve smoothing using different available filters in VO_2_FITTING.

**Figure 2:**
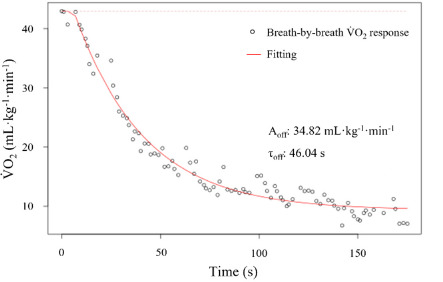
Representation of a recovery oxygen uptake ($\dot{V}$O_2_) to time curve response using VO_2_FITTING with the respective amplitude (A_off_) and time constant (τ_off_) identified, using the mono-exponential model without time delay.

$\dot{V}$O_2_ data as a function of time, obtained from the validation datasets, generated perfect fits, with the parameter estimates perfectly matching the known inputted values for all the four available models (standard error = 0; *p* < 0.001). An example of the different smoothing filters available in VO_2_FITTING and applied to the $\dot{V}$O_2_ post-exercise curve is presented in Figure 1. The mono-exponential models resulted in best fits during swimming and the recovery period for all swimmers, independently of the exercise intensity (Figure 2) since they were the only ones that fitted or because they presented a smaller standard error of regression and a residual sum of squares. Particularly for $\dot{V}$O_2_ off-transient kinetics data modelling, the mono-exponential function without TD had the best fit. Mean parameter estimates for all swimmers (individually fitted), standard deviations, and mean coefficients of variation are presented in Table 1. The coefficients of variation of the estimated A_on_, A_off_, τ_on_ and τ_off_ ranged between 1.4–2.7, 1.8–5.4, 18.1–26.0 and 5.6–15.1%, respectively, and 4.5–11.5% for TD_on_.

**Table 1:** Estimated $\dot{V}$O_2_ on- and off-transient kinetic parameters at the low, moderate, heavy and severe swimming intensity domains.

	Low	Moderate	Heavy	Severe	Low vs. Moderate		Moderate vs. Heavy		Heavy vs. Severe	
					*p*	ES (95% CI)	*p*	ES (95% CI)	*p*	ES (95% CI)	
$\dot{V}$O_2peak_ (mL•kg^−1^•min^−1^)	39.0 ± 6.1	42.2 ± 4.4	48.0 ± 3.4	51.4 ± 1.3	**0.03**	−0.8(−1.5 to 0.1)	**< 0.001**	−2.2 (−3.4 to −1.0)	**0.001**	−1.5(−2.4 to −0.6)	
EE$\dot{V}$O_2_(mL•kg^−1^•min^−1^)	38.8 ± 4.9	38.9 ± 8.1	46.3 ± 6.2	51.0 ± 8.4	0.97	−0.01(−0.6 to 0.6)	**0.006**	−1.1(−1.9 to −0.3)	0.07	−0.7(−1.3 to 0.1)	
*p*	0.91	0.06	0.45	0.81	-	-	-	-	-	-	
ES (95% CI)	0.04(−0.6 to 0.7)	0.7(−0.02 to 1.4)	0.3(−0.4 to 0.9)	0.1(−0.6 to 0.7)	-	-	-	-	-	-	
A_on_(mL•kg^−1^•min^−1^)	31.6 ± 3.9	33.2 ± 5.9	38.4 ± 5.6	40.3 ± 6.1	0.32	−0.3(−1.0 to 0.3)	**< 0.001**	−2.0(−3.1 to −0.9)	**0.01**	−1.0(−1.8 to −0.2)	
A_off_(mL•kg^−1^•min^−1^)	30.1 ± 5.8	29.9 ± 7.4	36.0 ± 5.7	40.3 ± 7.0	0.96	0.02(−0.6 to 0.6)	**0.03**	−0.8(−1.6 to −0.1)	0.05	−0.7(−1.4 to 0.01)	
*p*	0.49	0.13	0.28	0.99	-	-	-	-	-	-	
ES (95% CI)	0.2(−0.4 to 0.9)	0.5(−0.2 to 1.2)	0.4(−0.3 to 1.0)	−0.004(−0.6 to 0.6)	-	-	-	-	-	-	
τ_on_ (s)	15.8 ± 11.4	11.3 ± 2.3	13.9 ± 7.0	10.3 ± 4.6	0.18	0.5(−0.2 to 1.1)	0.17	−0.5(−1.1 to 0.2)	**0.04**	0.5(−0.2 to 1.1)	
τ_off_ (s)	30.8 ± 10.4	29.7 ± 8.4	28.7 ± 10.8	37.0 ± 9.2	0.82	0.1(−0.6 to 0.7)	0.85	0.06(−0.6 to 0.7)	0.08	−0.6(−1.3 to 0.07)	
*p*	**0.03**	**< 0.001**	**0.002**	**< 0.001**	-	-	-	-	-	-	
ES (95% CI)	−0.8(-1.5 to -0.1)	−2.5(−3.8 to −1.2)	−1.3(−2.2 to −0.4)	−2.7(−4.1 to −1.3)	-	-	-	-	-	-	
TD_on_ (s)	22.4 ± 6.6	19.8 ± 4.4	20.5 ± 3.4	19.6 ± 1.3	0.30	0.4(−0.3 to 1.0)	0.69	−0.1(−0.7 to 0.5)	0.44	0.3(−0.4 to 0.8)	

Abbreviations: $\dot{V}$O_2peak_: peak oxygen uptake; EE$\dot{V}$O_2_: end-exercise $\dot{V}$O_2_ value estimated using the off-transient kinetic model; A_on_ and A_off_, τ_on_ and τ_off_ and TD_on_: amplitude, time constant and time delay of the onset of the fast $\dot{V}$O_2_ component during swimming and recovery periods; ES: effect size; CI: confidence intervals

**Figure 3:**
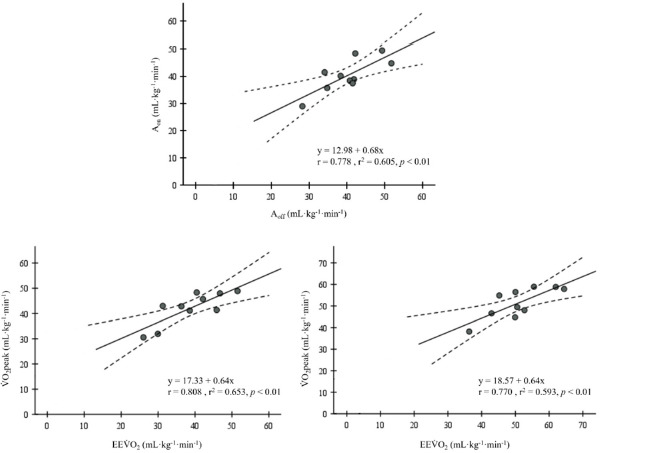
Relationships between on- and off-transient amplitude (A_on_ and A_off_) at severe, between peak $\dot{V}$O_2_ determined by the last 30 s of swimming ($\dot{V}$O_2peak_) and the end-exercise $\dot{V}$O_2_ determined by the fitting models and (EE$\dot{V}$O_2_) at moderate and severe intensity domains (upper and left and right lower panels, respectively).

The mean swimming velocities increased with the exercise intensity, corresponding to 1.18 ± 0.06, 1.24 ± 0.06, 1.31 ± 0.06 and 1.40 ± 0.06 m•s^−1^ for the low, moderate, heavy and severe domains (*p* < 0.001; ES [95% CI]: −2.6 [−4.0 to −1.3], −2.5 [−3.8 to −1.2] and −3.6 [−5.3 to −1.8], respectively). An increase in $\dot{V}$O_2peak_ with the intensity rise was observed, while both A_on_ and A_off_ increased from moderate to heavy and from heavy to severe intensities (*p* < 0.05). When analysing the on/off symmetry, the τ_on_ was lower than τ_off_ at all swimming intensity domains (*p* < 0.05). Significant correlations between on- and off-transient kinetic parameters were observed (Figure 3), with direct associations between A_on_ and A_off_ at severe and between $\dot{V}$O_2peak_ and EE$\dot{V}$O_2_ at moderate and severe intensity domains (*p* < 0.01).

## Discussion

Despite the importance of $\dot{V}$O_2max_ assessment (Duquette and Adam, 2024), $\dot{V}$O_2_ kinetic parameters’ interpretation is crucial to better understand the physiological response to a given effort (Jones and Poole, 2013). Although different commercial software is available to analyse $\dot{V}$O_2_ kinetic data, VO_2_FITTING is free and open-source, enabling an easier analysis and rapid feedback from continuous exercise, being a useful tool to monitor performance (Zacca et al., 2019). Our results obtained with the validation datasets demonstrated that VO_2_FITTING allowed to dynamically edit, process, filter and model also $\dot{V}$O_2_ post-exercise data with the available features commonly used in $\dot{V}$O_2_ kinetic modelling. The addition of this feature permits many advantages like allowing to compare $\dot{V}$O_2_ off-transient data with the respective previous exercise phase, with different assessments over time or with different exercise modes.

Breath-by-breath measurements have inherent non-uniformities in the breathing pattern, resulting in some variability around the mean $\dot{V}$O_2_ response, known as noise, that can produce some uncertainty in the estimation of the kinetic parameters (Keir et al., 2014; Lamarra et al., 1987). To improve this signal-to-noise ratio and have a clearer and more representative $\dot{V}$O_2_ profile, each participant usually performs several exercise transitions that are then time-aligned by interpolating to 1-s time intervals (Lamarra et al., 1987). This tool is available in VO_2_FITTING, both for on- and off-transient period analysis (Zacca et al., 2019). However, in the current study the incremental protocol was performed only once by each swimmer due to the complexity of the measurements in the aquatic environment. Thus, the estimation of different $\dot{V}$O_2_ kinetic parameters from a single transition was carried out using the bootstrapping method that provides reliable information about the estimated parameters (Curran-Everett, 2009; Millet and Borrani, 2009).

Increasing $\dot{V}$O_2peak_ mean values were observed along the swimming intensity domains spectrum (Fernandes et al., 2006; Monteiro et al., 2023), with the higher amplitude mean values (A_on_ and A_off_) occurring at heavy and severe compared to moderate and heavy exertions (respectively) being directly linked with the greater $\dot{V}$O_2peak_ mean values in these latter efforts. Regarding τ, it is a major focus of interest in the $\dot{V}$O_2_ kinetic related literature since it indicates the time needed to attain a $\dot{V}$O_2_ steady-state, having great importance from a practical perspective and being its accurate estimation highly relevant in the understanding of $\dot{V}$O_2_ kinetic on-response control (Carter et al., 2002). An invariant τ_on_ is related to a non-limiting oxygen delivery, at least until the heavy intensity domain, and control of muscle kinetics by intracellular processes (Carter et al., 2002; Grassi, 2000). The observed longer τ_off_ seems to be related to a slower rate of response towards reaching the $\dot{V}$O_2_ steady-state (Xu and Rhodes, 1999) that can be attributable to the external load on the thorax and increased airway resistance caused by the hydrostatic pressure from water immersion (Leahy et al., 2019), as to a lower muscle oxidative capacity related to the different body position adopted during swimming and recovery periods (Sousa et al., 2015). In addition, it seems that this kinetic parameter tends to remain constant along different intensities (Cleuziou et al., 2003), as it was evidenced in the current study.

Actually, the observed estimated coefficients of variation for A_on_, TD_on_, A_off_ and τ_off_ at the four different intensity domains were suitable, as previously reported (Zacca et al., 2019). τ_on_ presented slightly higher mean coefficient of variation values, which may be related to the natural variability of $\dot{V}$O_2_ response (Cooper and Garfinkel, 2022) and not to the constraints in the breathing pattern while swimming caused by the body position and the aquatic environment, as it was previously thought. In fact, when swimmers used the respiratory snorkel, a loss of synchronization was observed between the breathing pattern and the swimming movement, particularly at higher intensities, indicating that swimmers took advantage from the snorkel to breathe whenever they needed to (and not only when it was possible, as it occurs in free swimming) (Monteiro et al., 2023), approaching what takes place in other exercise modes (e.g., running or cycling).

There is a general consensus in the literature on a mono-exponential response for the low and moderate intensity domains (Cleuziou et al., 2003; Özyener et al., 2001; Poole and Jones, 2012), being consistent with the ideas that O_2_ debt matches the O_2_ deficit and of a linear control dynamics (Rossiter et al., 2005). Actually, the AnT is described as the point up to which there is no change or a small increase in [La^−^] and the $\dot{V}$O_2_ steady state is attained following the fast $\dot{V}$O_2_ response (Burnley and Jones, 2007; Pelarigo et al., 2017). Above the AnT there is a loss of body homeostasis, with higher participation of the anaerobic metabolism (Carvalho et al., 2020; Pelarigo et al., 2017). Thus, in the $\dot{V}$O_2_ kinetic related literature, heavy and severe intensity domains are commonly described by bi-exponential models (Cleuziou et al., 2003; Özyener et al., 2001; Sousa et al., 2015), where a delayed increase in $\dot{V}$O_2_ kinetics, known as a slow component, appears after approximately 2–3 min of exercise (Jones and Poole, 2013) as a sign of decreased efficiency of muscle contractions and of the recruitment of fibres with inherently slower $\dot{V}$O_2_ kinetics (Grassi et al., 2015; Zoladz et al., 2008).

At these higher intensities (above the AnT), an on/off symmetry is not always verified, contrary to what happens at low and moderate efforts (Özyener et al., 2001; Paterson and Whipp, 1991). However, the results of our experimental study did not evidence any second and delayed exponential increase during exercise nor recovery periods at the different swimming intensity domains. As a consequence of the swimming velocity rise along the incremental protocol, there was an evident decrease in the 200 m step duration (mean ± SD of 169.2 ± 8.0, 161.4 ± 8.1, 152.9 ± 7.0 and 142.8 ± 5.7 s for the low, moderate, heavy and severe intensities, respectively). On the one hand, and despite the 200 m step length validity and practical application during training sessions (Fernandes et al., 2011), the duration of the protocol steps may not have been sufficient to allow the development of the $\dot{V}$O_2_ slow component (Jones and Poole, 2013), suggesting that its emergence is closer to the third minute of exercise (Billat, 2000). On the other hand, the predominance of the aerobic component in the swimmers’ training sessions (Santos et al., 2024) may have decreased the $\dot{V}$O_2_ slow component due to an increased distribution of type I fibres and an increase in mitochondrial and capillary density (Billat, 2000; Holloszy and Coyle, 1984). In addition, the lack of isometric contractions in swimming and the effort distribution on all four limbs may suggest that no slow component should be expected (Demarie et al., 2001).

Despite the on/off symmetry observed in the present study, since the mono-exponential model presented the best fit, differences between both phases were found, particularly regarding TD. At the transition from rest to exercise, there must be a coordinated pulmonary, cardiovascular and muscular system response aiming to rapidly increase the O_2_ flux from the atmosphere to muscle mitochondria (Poole and Jones, 2012). Our results are in agreement with a commonly described delay of about 10–20 s, representing the time needed to O_2_ be unloaded in the muscle and the arrival of the same blood in the pulmonary vasculature for gas exchange (Barstow et al., 1996; Burnley and Jones, 2007). When the exercise ceases, a TD is often described but little interpreted and discussed, being possible to find a high range of mean values along the different exercise intensity domains (Billat et al., 2002; Cleuziou et al., 2003; Sousa et al., 2015).

However, the current results did not evidence a TD at the transition from exercise to recovery, at any of the studied intensity domains. Using VO_2_FITTING with breath-by-breath data without constraints and smoothing processes, it was possible to choose between different functions the one with the best fit and lower error. In this sense, raw $\dot{V}$O_2_ off-transient data with their inherent variability (Keir et al., 2014) demonstrated to be better characterized by a mono-exponential model without TD, indicating that the recovery period started immediately when swimmers stopped after each effort (Sousa et al., 2011) as it has been demonstrated at a muscle level (Behnke et al., 2009). The transition from the horizontal to the vertical position during the intervals and at the end of the protocol seems to facilitate the beginning of the recovery process (Leahy et al., 2019; Sousa et al., 2015), helping to explain the non-delay in our results.

Studies related to $\dot{V}$O_2_ kinetics at different exercise intensities and particularly focusing on the $\dot{V}$O_2_ slow component and off-transient phase have been carried out mainly in treadmill running and/or with a cycle ergometer (Cleuziou et al., 2003; Jones and Poole, 2013; Özyener et al., 2001), being this topic less studied in swimming. Despite the individual and cyclic characteristics of swimming (like running and cycling), the different environment conditions and the body position seem to affect the $\dot{V}$O_2_ kinetic response (Demarie et al., 2001; Leahy et al., 2019). With the use of VO_2_FITTING, and considering its availability to also analyse the $\dot{V}$O_2_ post-exercise data dynamically, we intend to contribute to future challenges, particularly to expand the knowledge about the on/off symmetry and $\dot{V}$O_2_ kinetics in such a peculiar exercise mode as swimming. In addition, future works including the comparison of the current methodological approach with only the $\dot{V}$O_2_ post-exercise data assessment, also allowing the inclusion of the undulatory underwater phases (Ruiz-Navarro et al., 2024), would add valuable information to this topic.

## Conclusions

VO_2_FITTING proved to be valid for characterizing $\dot{V}$O_2_ kinetics not only during continuous exercise, but also during the subsequent recovery period. With this free and open-source software applied for research and performance, it is possible to have rapid feedback about the $\dot{V}$O_2_ kinetics parameters without resorting to the use of complex mathematical programming. When characterizing $\dot{V}$O_2_ kinetics and the on/off symmetry at different swimming efforts, it seems that protocol steps of 200 m may not be sufficient to observe the development of the $\dot{V}$O_2_ slow component at higher intensity domains, particularly in high aerobically trained swimmers. In addition, due to the use of dynamic analysis software it was possible to observe that, after each effort, the recovery started immediately, a fact that could be more evident in swimming due to the body position and environment characteristics. We hope to encourage further research about these less studied topics, particularly in swimming.
